# High-fat diet-induced memory impairment in triple-transgenic Alzheimer's disease (3xTgAD) mice is independent of changes in amyloid and tau pathology^☆^

**DOI:** 10.1016/j.neurobiolaging.2014.02.010

**Published:** 2014-08

**Authors:** Elysse M. Knight, Isaura V.A. Martins, Sarah Gümüsgöz, Stuart M. Allan, Catherine B. Lawrence

**Affiliations:** Faculty of Life Sciences, The University of Manchester, Manchester, UK

**Keywords:** High-fat diet, 3xTgAD, Memory, Metabolism, Adipose tissue, Obesity

## Abstract

Obesity and consumption of a high-fat diet are known to increase the risk of Alzheimer's disease (AD). Diets high in fat also increase disease neuropathology and/or cognitive deficits in AD mouse models. However, the effect of a high-fat diet on both the neuropathology and memory impairments in the triple-transgenic mouse model of AD (3xTgAD) is unknown. Therefore, groups of 2-month-old male 3xTgAD and control (non-Tg) mice were maintained on a high-fat or control diet and memory was assessed at the age of 3–4, 7–8, 11–12, and 15–16 months using a series of behavioral tests. A comparable increase in body weight was observed in non-Tg and 3xTgAD mice after high-fat feeding at all ages tested but a significantly greater increase in epididymal adipose tissue was observed in 3xTgAD mice at the age of 7–8, 11–12, and 15–16 months. A high-fat diet caused memory impairments in non-Tg control mice as early as the age of 3–4 months. In 3xTgAD mice, high-fat consumption led to a reduction in the age of onset and an increase in the extent of memory impairments. Some of these effects of high-fat diet on cognition in non-Tg and 3xTgAD mice were transient, and the age at which cognitive impairment was detected depended on the behavioral test. The effect of high-fat diet on memory in the 3xTgAD mice was independent of changes in AD neuropathology as no significant differences in (plaques, oligomers) or tau neuropathology were observed. An acute increase in microglial activation was seen in high-fat fed 3xTgAD mice at the age of 3–4 months but in non-Tg control mice microglial activation was not observed until the age of 15–16 months. These data indicate therefore that a high-fat diet has rapid and long-lasting negative effects on memory in both control and AD mice that are associated with neuroinflammation, but independent of changes in beta amyloid and tau neuropathology in the AD mice.

## 1 Introduction

Alzheimer's disease (AD) is the most common form of dementia and is a significant health problem worldwide. AD is characterized by the presence of beta amyloid (Aβ) plaques and neurofibrillary tangles within the brain and patients present with cognitive deficits including impairments in learning and memory. The occurrence of AD is mostly sporadic affecting individuals over the age of 65 years. However, there are several factors that can increase AD risk including diabetes, stroke, atherosclerosis, and obesity and/or metabolic syndrome.

Obesity is a major health problem associated with increased risk of several diseases such as diabetes. However, obesity at midlife can also increase the risk of dementia and AD later in life (Beydoun et al., 2008; Fitzpatrick et al., 2009; Gustafson et al., 2003; Hassing et al., 2009; Kivipelto et al., 2005; Profenno et al., 2009; Rosengren et al., 2005; Whitmer et al., 2005, 2007, 2008), an effect that is independent of the conditions associated with obesity that are also risk factors for AD, such as type 2 diabetes and cardiovascular disease (Hassing et al., 2009; Whitmer et al., 2005, 2007). This relationship between obesity and AD appears to depend on age as obesity can decrease the risk of AD in later life (Fitzpatrick et al., 2009) and weight loss actually precedes disease onset (Buchman et al., 2005; Stewart et al., 2005). Obesity is often caused by and is associated with, consumption of diets that are high in fat. The prevalence of AD is greater in countries with higher intake of high fat and/or calorie diets but lower in those that consume diets low in fat (Grant, 1997; Panza et al., 2004). Furthermore, epidemiologic studies suggest diets high in saturated fats (especially in midlife) are a major risk factor for the development of AD (Eskelinen et al., 2008; Grant, 1999; Kalmijn et al., 1997; Laitinen et al., 2006; Luchsinger et al., 2002), and this risk is higher in individuals with the APOE ε4 allele (Laitinen et al., 2006; Luchsinger et al., 2002).

Disease neuropathology and/or behavioral deficits are enhanced in mouse models of AD that are maintained on a high-fat diet (without high levels of cholesterol) (Herculano et al., 2013; Ho et al., 2004; Julien et al., 2010; Maesako et al., 2012a; Pedrini et al., 2009; Phivilay et al., 2009). There are several well-characterized mouse models of AD, most with mutations in amyloid precursor protein (APP) and/or presenilin 1/2 (PS1/2) that present with Aβ plaques only. The triple-transgenic AD (3xTgAD) mouse has mutations in APP_Swe_, PS1_M146V_, and tau_P301L_, and as a consequence, develops temporal- and region-specific Aβ plaques and tangle-like pathology that closely resemble the pathology seen in the human AD brain, in addition to developing functional impairments, including learning and memory deficits (Billings et al., 2005; Oddo et al., 2003a, 2003b). The 3xTgAD mouse does not present with an aggressive pathology, as we do not observe Aβ plaques and tangle-like pathology until approximately at the age of 12 months, which is after cognitive deficits are detected (Billings et al., 2005; Knight et al., 2012, 2013). The 3xTgAD mouse therefore allows us to identify the effect of a high-fat diet before significant AD neuropathology, and to also study the relationship between Aβ plaques and tau.

In humans, the severity of AD-related neuropathology especially Aβ plaque burden, does not always correlate with, or is predictive of, cognitive deficits, and memory impairments can occur in mouse AD models in advance of overt Aβ plaque (and tangle) pathology (Billings et al., 2005; Oddo et al., 2003b; Serrano-Pozo et al., 2011). Most studies to date examining the role of high-fat diets in AD mouse models have assessed neuropathology only (Julien et al., 2010; Pedrini et al., 2009; Phivilay et al., 2009) and few have monitored both neuropathology and memory (Herculano et al., 2013; Ho et al., 2004; Maesako et al., 2012a). Furthermore, most of these studies identifying an effect of a high-fat diet in AD on neuropathology and behavior have modified diet in AD mice only and have not studied the effect of diet in control animals (Ho et al., 2004; Maesako et al., 2012a). As high-fat diets have been shown to affect memory in cognitively-normal rodents (McNeilly et al., 2011; Pistell et al., 2010; Winocur and Greenwood, 2005), it is not clear whether the cognitive deficits observed in AD mice fed a high-fat diet are related to or independent of AD pathology. Finally, most reports on the changes in cognition and/or neuropathology in high-fat fed AD mice have studied just one time point, and thus only report effects at a single stage and/or severity of the disease. It is possible that some of the effects of a high-fat diet in AD might be transient, which will be missed in such studies.

The aim of this study therefore was to characterize longitudinally the impact of a high-fat diet on both cognition and neuropathology in male 3xTgAD and non-transgenic (non-Tg) control mice. Memory was assessed using a battery of behavioral tests. No study to date has compared the effects of a high-fat diet on both cognition and neuropathology in 3xTgAD mice and we show that a high-fat diet impairs memory in both the non-Tg control and 3xTgAD mice, the effects of which depend on the behavioral test used and duration of diet. Effects of high-fat diet on cognition in the 3xTgAD mice occurred without any significant effect on AD neuropathology.

## 2 Methods

### 2.1 Animals and diet

Male 3xTgAD mice expressing mutant PS1_M146V_, APP_Swe_, Tau_P301L_, and control non-Tg (129/C57BL6) mice were originally supplied by Frank LaFerla (Irvine, CA, USA) (Oddo et al., 2003b) and an in-house colony established in Manchester. All mice were kept in standard housing conditions (humidity 50%–60%, temperature 21 ± 1 °C, 12:12 hour light-dark cycle with lights on at 07:00 hours) and given *ad libitum* access to standard rodent chow and water unless stated. All animal experiments were carried out in accordance with the United Kingdom Animals (Scientific Procedures) Act 1986. At the age of 8 weeks 3xTgAD and non-Tg control mice were placed on either a high-fat diet (60% energy from fat, 35% fat content by weight, 13% saturated fatty acids, 58G9, Test Diets, supplied by IPS Product Supplies Ltd, UK) or control diet (12% energy from fat, 5% fat content by weight, 0.78% saturated fatty acids, 58G7). Separate groups of mice were maintained on their respective diets until the age of 3–4 (n = 10–12/group), 7–8 (n = 10–11/group), 11–12 (n = 9–10/group), or 15–16 (n = 6–10/group) months when behavioral tests were performed. Body weight was monitored in all mice from weaning until behavioral assessment. There were a few deaths because of unknown causes over the cause of the study, and these animals were not included in the analyses (7–8 months: non-Tg control n = 1; 11–12 months: 3xTgAD control n = 1, 3xTgAD high-fat n = 1; 15–16 months: non-Tg high-fat n = 3, 3xTgAD control n = 2, 3xTgAD high-fat n = 1).

### 2.2 Behavioral tests

Male non-Tg control and 3xTgAD mice were subjected to the Y-maze spontaneous alternation, smell recognition, novel object recognition, and Morris water maze (MWM) tests. On the days of behavioral evaluation, home cages were placed in the testing room 30 minutes before testing to allow habituation. All behavioral observations were made between 1000 hours and 1600 hours. The order of observation during this period was randomized across animals and all subsequent analysis was performed blinded to genotype and diet. No more than one behavioral test was completed during any single day. All equipment was cleaned between animals.

#### 2.2.1 Y-maze spontaneous alternation test

Short-term working memory was assessed in the Y-maze spontaneous alternation test using a black opaque Perspex Y-maze with 3 arms (A, B, and C) each containing a visual cue (arm dimensions; 15 cm × 10 cm × 10 cm). Each animal was placed in turn in arm A of the Y-maze and allowed to explore for 8 minutes and the arm entries made by each animal were recorded. Arm entry was defined as having all 4 paws in the arm. Spontaneous alternation was defined as a successive entry into 3 different arms, on overlapping triplet sets (Hiramatsu et al., 1997; Wall and Messier, 2002). The percentage number of alternations was calculated as the number of actual alternations divided by the maximum number of alternations (the total number of arm entries minus 2). The total number of moves was also recorded as an index of ambulatory activity (Hiramatsu et al., 1997).

#### 2.2.2 Smell recognition test

Short-term non-associative memory based on the natural exploration of novelty in mice was assessed in the smell recognition test. All mice were habituated to a black opaque polycarbonate circular arena (diameter, 30 cm × height, 21 cm) for 5 minutes over 2 days. On the day of testing, mice were placed in the arena and allowed to explore 2 identical unfamiliar scented balls for 10 minutes (phase 1). The scented balls were placed in the center of the arena, 5 cm from the edge and 8 cm away from each other. The hollow balls (Chad Valley, UK) were filled with cotton wool and 0.5 mL of scent (orange, lemon, vanilla, or almond, Dr Oetker Ltd, UK) was evenly distributed into the balls via small holes. Mice were then removed, one of the balls was replaced with a novel scented ball, and after a delay of 3 minutes, mice were placed back into the arena and allowed to explore for a further 4 minutes (phase 2). The novel scented ball was placed randomly in either the left or right position. All behavior was recorded with a camera (Sanyo Xacti VPC-C4, SANYO Fisher, CA, USA) and MP4 video-clips were converted to an AVI format using Pazera MP4 to AVI converter 1.3 (Pazera-Software, PL). The duration (seconds) spent exploring the scented balls was then measured using Observer 5.0 software (Noldus, Wageningen, the Netherlands). Exploration was defined as the amount of time the animals spent with their nose pointing within 2 cm of the scented balls. The percentage time spent exploring the scented balls was calculated for phases 1 and 2.

#### 2.2.3 Novel object recognition test

Short-term non-associative memory based on the natural exploration of novelty in mice was also assessed in the novel object recognition test. The task was performed the same as the smell recognition task, but during phase 1 of the task the mice were placed in the arena and allowed to explore 2 identical unfamiliar wooden painted trial objects (Universe of Imagination, Geoffrey, Inc, UK) for 10 minutes. Mice were then removed and one of the objects was replaced with a novel object that varied in shape and color. After an interval of 1 hour, mice were allowed to explore the familiar object and the novel object for 4 minutes. The time spent exploring the familiar and novel objects was calculated for phases 1 and 2 as for the smell recognition test.

#### 2.2.4 MWM

Spatial reference memory was assessed using the MWM using a 1.2 m diameter (height, 25 cm) circular white opaque plastic tank that contained water maintained at a temperature of 21 °C–22 °C and made opaque using water-soluble nontoxic white paint (Universe of Imagination, Geoffrey Inc, UK). During the MWM test, mice were given 2 days of visual platform training followed by 8 days of hidden platform training and a 1 day probe trial (based on protocol by Vorhees and Williams (2006)). Briefly, for the acquisition of the visual platform training, mice were placed into the maze without spatial cues, and allowed to locate a visual flagged platform. If the platform was not found within 2 minutes, the mouse was gently guided to it. Mice were given 4 trials each day for 2 days with a different start position and flagged platform location each trial. For the acquisition of the hidden platform test, 4 trials per day were conducted for 8 days. The sequence of start positions was different on each training day and visual spatial cues were located outside the tank. The latency to find the platform was recorded with a maximum of 2 minutes allowed. To test memory retention of the platform location, mice underwent a probe trial 24 hours after the final hidden platform training trial. During the probe trial, the platform was removed, and the mouse was placed in the pool and allowed to swim for 30 seconds. Time spent in each quadrant was recorded. Each trial was monitored and analyzed using a CCTV tracking camera (Vista protos IV, UK) and 2020 PLUS tracking software (HVS Image, Buckingham, UK). The escape latency (second) during both visual platform and hidden platform training and the percentage time in the target quadrant during the probe trial and swim speed (meter/second) were calculated.

### 2.3 Tissue preparation

After the behavioral tests all animals were terminally anesthetized with 3.5% isoflurane (30% O_2_ and 70% N_2_O) and 0.9% saline was perfused-transcardially. The brain was rapidly removed and one hemisphere immerse-fixed in 4% paraformaldehyde at room temperature for 24 hours. This hemisphere was then cryoprotected in 30% sucrose (in 0.1 M phosphate buffer [PB]) at 4 °C for 24 hours before being frozen in isopentane on dry ice. The hippocampus was dissected from the other hemisphere of the brain and frozen on dry ice. All samples were stored at −80 °C until assay. One epididymal fat pad was also dissected and weighed.

#### 2.3.1 Immunohistochemistry

Coronal 30 μm brain sections were cut (from −1.34 mm to −3.88 mm relative to bregma according to the atlas of Paxinos and Franklin (2001)), on a freezing sliding microtome (Bright 8000-001, Bright Instrument Co Ltd, UK). Immunohistochemistry for either Aβ or phosphorylated tau was then performed on free-floating brain sections. Briefly, endogenous peroxidase was removed before treatment in blocking solution (10% normal horse serum in PB/0.3% triton). Sections were then incubated at 4 °C overnight with either a mouse monoclonal anti-human amyloid 6E10 (1:3000, Covance-Signet Laboratories, UK) for Aβ or mouse monoclonal anti-human PHF-tau (AT8; 1:1000, Autogen Bioclear, UK) for hyperphosphorylated tau. After washes in PB/0.3% triton, sections were treated for 2 hours in a biotinylated horse anti-mouse IgG antibody (1:500; Vector Laboratories Ltd, Peterborough, UK). Following washes (in 0.1 M PB), sections were immersed in avidin-biotin-peroxidase complex (ABC, Vector Laboratories Ltd) for 30 minutes, rinsed in 0.1 M PB and color-developed using a 0.05% diaminobenzidine solution (in 0.01% H_2_O_2_). Sections were mounted onto gelatin-coated slides, dried, and coverslipped before viewing under a light microscope. The number of immunopositive cells (neurons) expressing tau was counted unilaterally, using a light microscope, throughout the hippocampus. The average number of cells per section was calculated and the group mean determined. The Aβ plaque burden was determined throughout in the hippocampus. The plaque area was measured in each section and the plaque burden calculated as average plaque area/section (μm^2^).

To detect microglia, immunohistochemistry was performed as previously mentioned but using a rabbit anti-Iba1 primary antibody (1:2000, Wako Chemicals, Germany) and a biotinylated goat anti-rabbit IgG antibody (1:500; Vector Laboratories Ltd), all in 2% normal goat serum in PB/0.3% triton. Activated microglia throughout the hippocampus were identified according to previous studies. Drake et al., 2011 defined by either an increase in Iba1 staining; enlarged cell bodies; complete or partial loss of thin elongated process. The number of activated microglia per section was counted and the average calculated. For all immunohistochemical analyses the investigator was blinded to genotype and diet.

#### 2.3.2 Aβ oligomer enzyme-linked immunosorbent assay

The hippocampus of control and high-fat fed 3xTgAD mice, at all ages tested, were prepared by homogenization in extraction buffer (50 mM Tris-Cl, 150 mM NaCl, 1% CHAPS, pH 7.6, containing proteases inhibitors) and samples were left to stand for 3 hours. The homogenates were then centrifuged at 15,000 rpm at 4 °C for 30 minutes. Human Aβ oligomers were analyzed in the hippocampal supernatant by enzyme-linked immunosorbent assay (ELISA) (IBL International, Germany) according to manufacturer's instructions. The ELISA uses mouse monoclonal anti-human Aβ (N) (82E1) antibodies that recognize the N-terminus of human Aβ specifically, with 2 or more epitopes. The level of oligomers in the hippocampus of non-Tg control mice was not tested, as no human oligomers are detected in the brain of these mice (data not shown).

### 2.4 Statistical analysis

Data are represented as mean ± standard error of the mean. Epididymal fat weight, evaluation of memory in the spontaneous alternation test, and the number of activated microglia were examined using a 2-way analysis of variance (ANOVA) with Bonferroni post hoc analysis and for the smell recognition, novel object tests, and neuropathology a Student *t* tests was used. Morris water maze training was assessed between cohorts on individual training days and compared within cohorts between the first day of training and successive days of training to assess improvement over time via 3-way repeated measures ANOVAs with Scheffe post hoc analysis, as was body weight. The probe test was analyzed using a 2-way ANOVA with Bonferroni post hoc analysis. Statistical significance was taken at *p* < 0.05.

## 3 Results

### 3.1 High-fat diet increased body weight and fat mass in both non-Tg and 3xTgAD mice

**Fig. 1:**
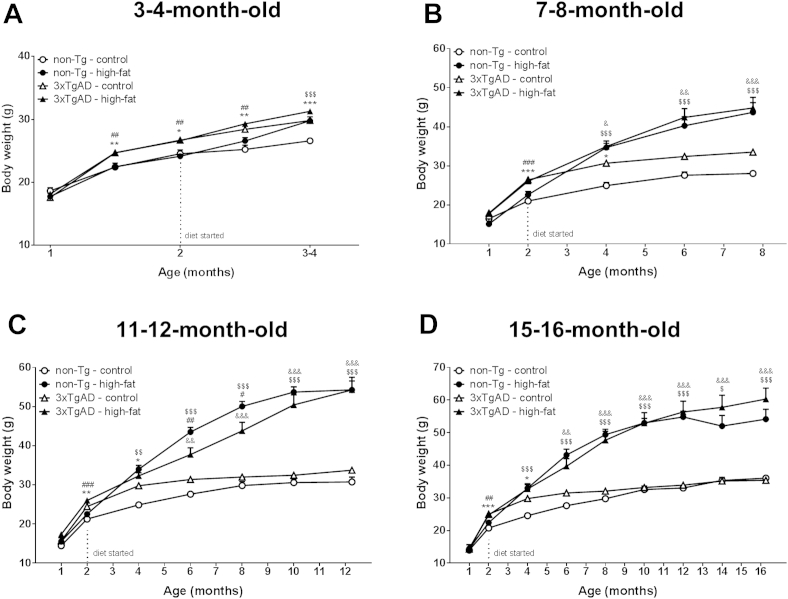
Body weight of 3xTgAD and non-Tg mice in response to a high-fat diet. Male 3xTgAD and non-Tg control mice were maintained on either a control or high-fat diet from the age of 8 weeks (at the age of 2 months) and body weight was assessed in separate groups of mice at the age of 3–4 (A), 7–8 (B), 11–12 (C), and 15–16 (D) months. Data are means ± SEM, n = 6–12/group. * *p* < 0.05, ** *p* < 0.01, *** *p* < 0.001 non-Tg versus 3xTgAD mice on control diet; ^##^*p* < 0.01, ^###^*p* < 0.001 non-Tg versus 3xTgAD mice on high-fat diet; ^$^*p* < 0.05, ^$$^*p* < 0.01, ^$$$^*p* < 0.001 non-Tg on a control versus high-fat diet; ^&^*p* < 0.05, ^&&^*p* < 0.01, ^&&&^*p* <0.001 3xTgAD on a control versus high-fat diet. Three-way repeated measures ANOVAs with Scheffe post hoc analysis. Abbreviations: ANOVA, analysis of variance; SEM, standard error of the mean.

For all groups of mice at the age of 1 month, there was no significant difference in body weight between non-Tg and 3xTgAD mice (Fig. 1). By the age 2 months, before modifying diet, 3xTgAD mice weighed significantly (*p* < 0.05–0.001) more than non-Tg control mice in all groups.

At the age of 3–4 months, non-Tg mice on a high-fat diet were significantly (*p* < 0.001; Fig. 1A) heavier than those maintained on a control diet, but no difference was observed between 3xTgAD mice on a control versus high-fat diet. As at the age of 2 months, 3 to 4-month-old 3xTgAD mice weighed significantly (*p* < 0.001) more than non-Tg mice when on a control diet. At the age of 7–8, 11–12, and 15–16 months, both 3xTgAD and non-Tg mice on a high-fat diet weighed more than their respective control fed mice, and there was now no difference in body weight between control-fed non-Tg and 3xTgAD mice (Fig. 1B–D).

At all ages tested male 3xTgAD mice on a control diet displayed no difference in epididymal fat pad weight compared with the control fed non-Tg mice (Table 1). High-fat feeding led to a significant (*p* < 0.001) increase in epididymal fat pad weight in both 3xTgAD and non-Tg mice at the age of 3–4 and 7–8 months compared with their respective control fed mice. Epididymal fat pad weight was significantly (*p* < 0.01–0.001) lower in the 3xTgAD than the non-Tg mice on a high-fat diet at the age of 3–4 months but higher in 3xTgAD mice at the age of 7–8 months. At the age of 11–12 and 15–16 months, epididymal fat pad weight was not different in non-Tg mice on a high-fat diet, whereas it was significantly (*p* < 0.001) higher in the 3xTgAD mice on a high-fat diet compared with their respective control fed mice.

### 3.2 High-fat diet impaired memory in non-Tg and 3xTgAD mice

#### 3.2.1 Y-maze

**Fig. 2:**
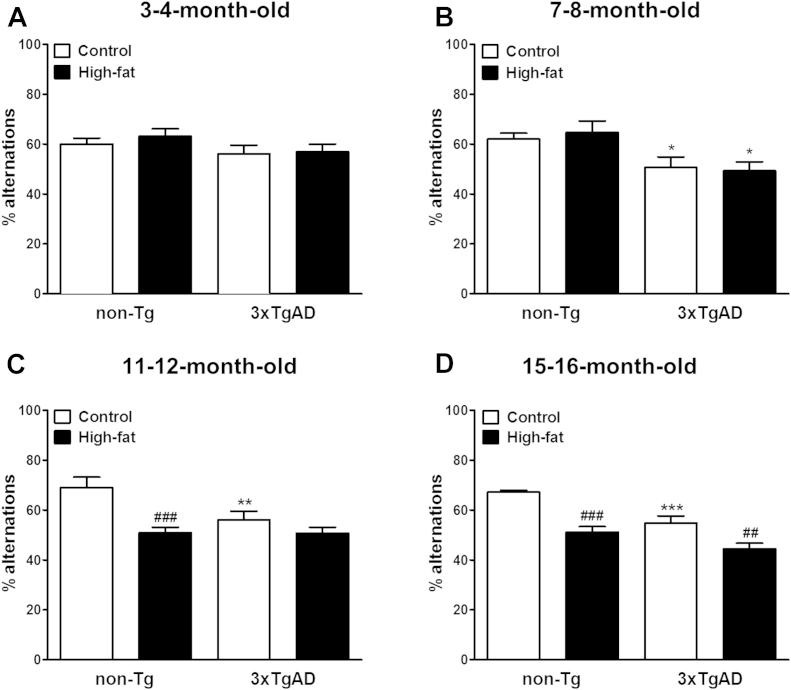
High-fat diet impairs learning in the Y-maze spontaneous alternation test in 3xTgAD and non-Tg mice. Male 3xTgAD and non-Tg control mice were maintained on either a control or high-fat diet from the age of 8 weeks. Cognitive function was assessed in the Y-maze spontaneous alternation test in separate groups of mice at the age of 3–4 (A), 7–8 (B), 11–12 (C), and 15–16 (D) months. Data are means ± SEM. n = 6–12/group. * *p* < 0.05, ** *p* < 0.01, *** *p* < 0.001 versus non-Tg mice on the same diet, ^##^*p* < 0.01, ^###^*p* < 0.001 versus control-fed mice of the same genotype. Two-way ANOVA with Bonferroni post hoc analysis. Abbreviations: ANOVA, analysis of variance; SEM, standard error of the mean.

At the age of 3–4 months, there was no significant effect of genotype or diet on memory performance in the Y-maze (Fig. 2A). By the age of 7–8 months, 3xTgAD mice showed a diet-independent decrease (18% and 24%; *p* < 0.05) in percentage alternations compared with non-Tg mice (Fig. 2B). Significantly, fewer percentage alternations (19%; *p* < 0.01) were still detected in 11 to 12-month-old control fed 3xTgAD compared with non-Tg mice (Fig. 2C). At the age of 11–12 months, high-fat diet impaired memory (26% reduction; *p* < 0.001) in non-Tg mice. However, a high-fat diet did not affect memory in 11 to 12-month-old 3xTgAD mice. At the age of 15–16 months, 3xTgAD mice on a control diet performed fewer percentage alternations (19% *p* < 0.001; Fig. 2D) than non-Tg mice. A high-fat diet not only impaired memory in 15 to 16-month-old non-Tg mice but also affected cognition in 3xTgAD mice as less percentage alternations were observed in high-fat compared with control fed mice for both genotypes (24% and 19%; *p* < 0.001 and *p* < 0.01 for non-Tg and 3xTgAD, respectively). The number of moves was transiently decreased in 3xTgAD mice at the age of 3–4 and 7–8 months but at all ages tested, a high-fat diet had no effect on the total number of moves in both non-Tg and 3xTgAD mice (data not shown).

#### 3.2.2 Smell recognition

**Fig. 3:**
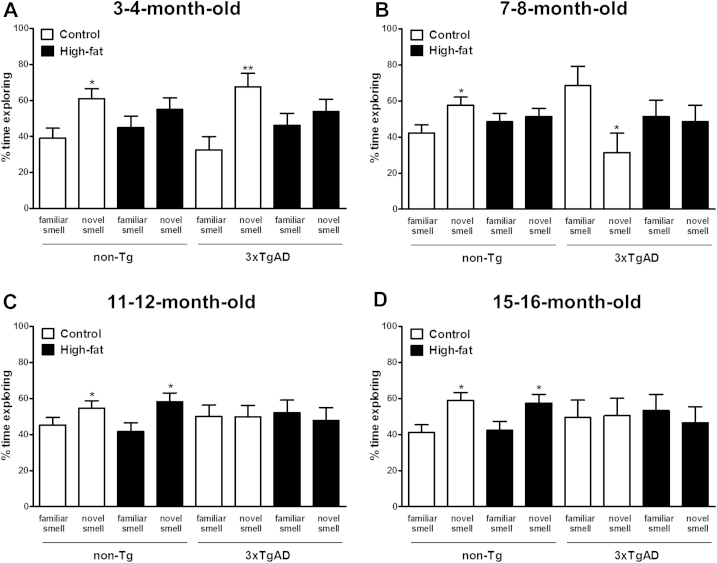
High-fat diet impairs learning in the smell recognition test in 3xTgAD and non-Tg mice. Male 3xTgAD and non-Tg control mice were maintained on either a control or high-fat diet from the age of 8 weeks. Cognitive function was assessed in the smell recognition test in separate groups of mice at the age of 3–4 (A), 7–8 (B), 11–12 (C), and 15–16 (D) months. During phase 1, the mice were placed in an arena for 10 minutes with 2 identically scented balls (data not shown). During phase 2, after an interval of 3 minutes, the mice were placed back into the arena with one familiar scented ball (presented in phase 1) and one novel scented ball for 4 minutes and percentage time exploring balls was compared. Data are means ± SEM. n = 6–12/group. * *p* < 0.05, ** *p* < 0.01 for novel versus familiar smell; Student *t* test. Abbreviation: SEM, standard error of the mean.

At the age of 3–4, 7–8, 11–12, and 15–16 months, there was no difference in exploration of identical scented balls during phase 1 of the smell recognition test for all groups of mice (data not shown). During phase 2, after a delay of 3 minutes, non-Tg mice at all ages on a control diet spent a significantly (*p* < 0.05) higher percentage of time exploring the novel scented ball than the familiar scented ball indicating memory formation (Fig. 3A–D). Memory was unimpaired in 3xTgAD mice on a control diet at earlier time points as mice spent more time exploring the novel scented ball at the age of 3–4 months (*p* < 0.01) and more time exploring the familiar scented ball at the age 7–8 months (*p* < 0.05). However, memory was impaired in control fed 3xTgAD mice at the age of 11–12 and 15–16 months as no difference in exploration between the novel and familiar smell was observed. High-fat feeding transiently impaired memory in non-Tg mice as no preference for exploring the novel or familiar scented ball was detected at the age of 3–4 and 7–8 months but by the age of 11–12 and 15–16 months high-fat fed non-Tg mice spent more time exploring the novel scented ball (*p* < 0.05). In contrast, the 3xTgAD mice on a high-fat diet showed impaired memory at all ages, as they did not show a preference for the novel or familiar smell.

#### 3.2.3 Novel object recognition

**Fig. 4:**
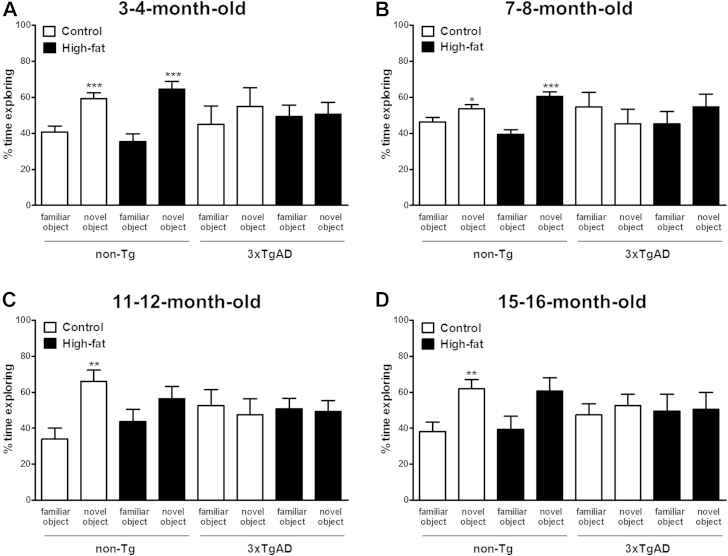
High-fat diet impairs learning in the novel object recognition test in non-Tg mice. Male 3xTgAD and non-Tg control mice were maintained on either a control or high-fat diet from the age of 8 weeks. Cognitive function was assessed in the novel object recognition test in separate groups of mice at the age of 3–4 (A), 7–8 (B), 11–12 (C), and 15–16 (D) months. During phase 1, the mice were placed in an arena for 10 minutes with 2 identical novel objects (data not shown). During phase 2, after an interval of 1 hour, the mice were placed back into the arena with one familiar object (presented in phase 1) and one novel object for 4 minutes and percentage time exploring objects was compared. Data are means ± SEM. n = 6–12/group. * *p* < 0.05, ** *p* < 0.01, *** *p* < 0.01 for novel versus familiar object; Student *t* test. Abbreviation: SEM, standard error of the mean.

At the age of 3–4, 7–8, 11–12, and 15–16 months, there was no difference in exploration of identical objects during phase 1 of the test in both non-Tg and 3xTgAD mice (data not shown). At the age of 3–4 and 7–8 months, during phase 2 after an interval of 1 hour, non-Tg mice on a control or high-fat diet spent significantly (*p* < 0.05 and *p* < 0.001) more time exploring the novel versus the familiar object (Fig. 4A and B). At the age of 11–12 and 15–16 months, the non-Tg mice on a control diet also spent significantly more time exploring the novel versus the familiar object (*p* < 0.01, Fig. 4C and D), whereas the high-fat diet impaired memory at these ages. In 3xTgAD mice, memory was impaired at all ages on both control and high-fat diet (Fig. 4A–D).

#### 3.2.4 Morris water maze

By the second day of visual platform training in the MWM there was no significant difference in escape latency between any group at the age of 3–4, 7–8, 11–12, or 15–16 months (data not shown).

**Fig. 5:**
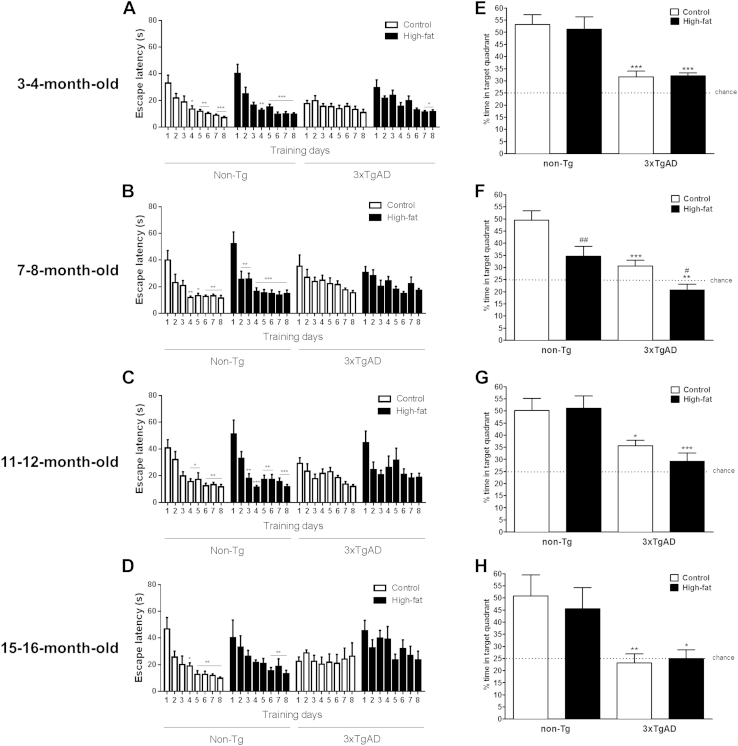
High-fat diet impairs learning in the Morris water maze in 3xTgAD and non-Tg mice. Male 3xTgAD and non-Tg control mice were maintained on either a control or high-fat diet from the age of 8 weeks. Cognitive function was assessed in the Morris water maze in separate groups of mice at the age of 3–4 (A), 7–8 (B), 11–12 (C), and 15–16 (D) months. Mice were given 4 trials a day for 8 days of submerged platform training in the MWM (A–D). Twenty-four hours after the final trial the mice were given a probe test with no platform (E–H). Data are mean +/− SEM. For escape latency, * *p* < 0.05, ** *p* < 0.01, *** *p* < 0.001 day 1 versus days 2–8. For percentage time in target, * *p* < 0.05, ** *p* < 0.01, *** *p* <0.001 versus non-Tg mice on the same diet, ^#^*p* < 0.05, ^##^*p* < 0.01 versus control-fed mice of the same genotype. Abbreviations: MWM, Morris water maze; SEM, standard error of the mean.

During hidden platform training (Fig. 5A–D), non-Tg mice on a control diet showed significantly decreasing escape latency over training days at all ages tested (days 2–8 versus day 1; *p* < 0.05–0.001), whereas the 3xTgAD mice on a control diet showed no evidence of learning. On a high-fat diet, non-Tg mice got quicker over training days at the age of 3–4, 7–8, 11–12, and 15–16 months (days 2–8 versus day 1; *p* < 0.05–0.001), whereas the 3xTgAD mice on a high-fat diet, got quicker only at the age of 3–4 months (days 7–8 versus day 1; *p* < 0.05) yet were unable to learn at the age of 7–8, 11–12, and 15–16 months. On individual training days there was no significant difference in escape latency between the cohorts.

At all ages tested during the probe test, 3xTgAD mice on either a control or high-fat diet spent significantly less time in the target quadrant when compared with their respective fed non-Tg mice (*p* < 0.05–0.001, Fig. 5E–H). High-fat feeding had no effect on memory in 3 to 4-month-old 3xTgAD or non-Tg mice. However, by the age of 7–8 months, a high-fat diet impaired memory as a decrease (30%–32%) in the time spent in the target quadrant was observed in both high-fat fed 3xTgAD and non-Tg mice when compared with control fed mice. Effects of high-fat diet on memory were transient as at the age of 11–12 and 15–16 months no difference in time spent in the target quadrant was observed between 3xTgAD or non-Tg mice on either a control or high-fat diet. During the probe test at the age of 3–4 months swim speed was significantly higher in 3xTgAD mice compared with non-Tg control mice on either diet (non-Tg, control 0.23 ± 0.01 m/s, high-fat 0.24 ± 0.01 m/s; 3xTgAD, control 0.29 ± 0.01 m/s, high-fat 0.29 ± 0.01 m/s; *p* < 0.001, 3xTgAD versus non-Tg on either diet). At all other ages (7–8, 11–12, and 15–16 months) there was no longer an effect of genotype on swim speed. There was no effect diet on swim speed in all groups of mice at all ages (data not shown).

### 3.3 High-fat diet had no effect on Aβ and tau pathology in 3xTgAD mice

**Fig. 6:**
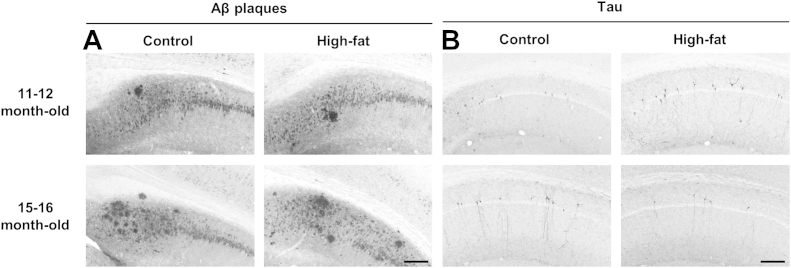
High-fat diet has no effect on Aβ or tau neuropathology in the hippocampus of 3xTgAD mice. Male 3xTgAD and non-Tg control mice were maintained on either a control or high-fat diet from the age of 8 weeks until the age of 11–12 and 15–16 months. Immunohistochemistry for Aβ (A) or hyperphosphorylated tau (B) was performed using 6E10 and AT8 antibodies, respectively. Representative sections for 3xTgAD mice are shown for the hippocampus indicating the extracellular Aβ plaque burden and tau-positive neurons. Quantification is presented in Table 2. Scale bars 200 μm. Abbreviation: Aβ, beta amyloid.

To assess the effect of a high-fat diet on Aβ peptide deposition, sections from all mice were stained with 6E10, an antibody that recognizes amino acid residue 1–16 of beta-amyloid. No extracellular Aβ plaques were detected in the brains of control or high-fat fed 3xTgAD mice at the age of 3–4 and 7–8 months but by the age of 11–12 months, occasional Aβ plaques were detected in the hippocampus of 3xTgAD mice that were more prevalent at the age of 15–16 months (Table 2 and Fig. 6A). A high-fat diet had no effect on the Aβ plaque burden in the hippocampus of 3xTgAD mice at the age of 11–12 or 15–16 months (Table 2 and Fig. 6A). No extracellular Aβ plaques were detected in the brains of non-Tg mice or in the cortex and amygdala of 3xTgAD mice on either a control or high-fat diet.

No cells positive for hyperphosphoryated tau were found in the brain of 3 to 4-month-old 3xTgAD mice. At the age of 7–8, 11–12, and 15–16 months, tau-positive cells were detected in the hippocampus and amygdala. In all brain regions, there was no significance difference in the number of tau-positive cells between 3xTgAD mice fed a control or high-fat diet (Table 2 and Fig. 6B). No hyperphosphoryated tau was detected in the brains of non-Tg mice or in the cortex of 3xTgAD mice on either a control or high-fat diet.

The level of Aβ oligomers was analyzed in the hippocampus of 3xTgAD mice by ELISA. There was no difference in the amount of oligomers detected between control and high-fat fed 3xTgAD mice at all ages tested (Table 2).

### 3.4 High-fat diet increases microglia activation in non-Tg and 3xTgAD mice

**Fig. 7:**
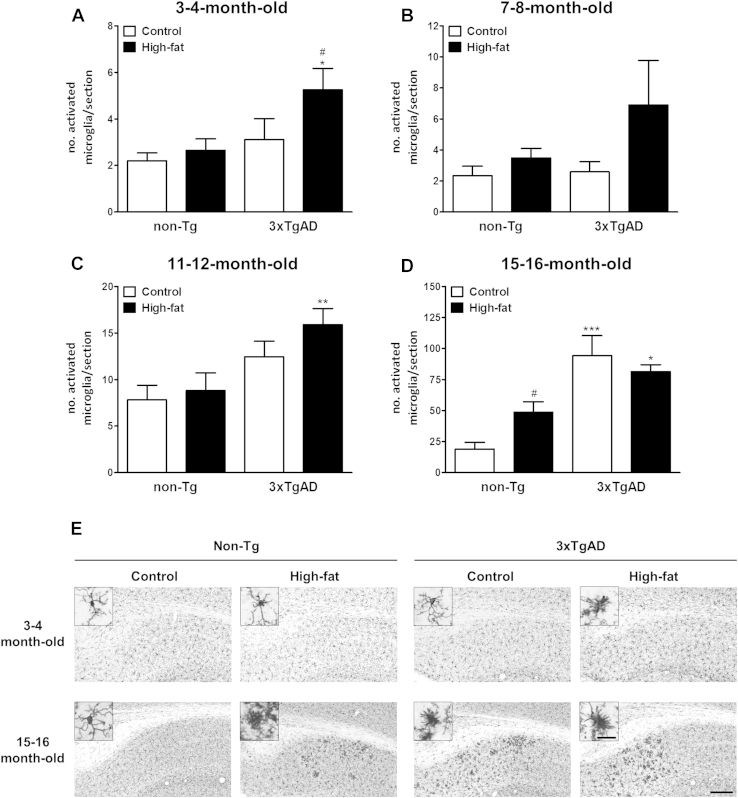
High-fat diet increases microglia activation in the hippocampus of 3xTgAD and non-Tg mice. Male 3xTgAD and non-Tg control mice were maintained on either a control or high-fat diet from the age of 8 weeks until the age of 3–4 (A), 7–8 (B), 11–12 (C), and 15–16 (D) months. Microglia activation were identified by increased Iba1 immunopositivity, enlarged and/or irregular cell bodies, thickened, partial or complete loss of processes and were seen after high-fat feeding in 3xTgAD mice at the age of 3–4 months and non-Tg mice at the age of 15–16 months. An increase in microglia activation was also observed in control fed 3xTgAD mice over time. (E) Representative photomicrographs in the hippocampus from 3–4 and 15 to 16-month-old mice. Scale bars 200 μm and 25 μm (insets). * *p* < 0.05, ** *p* < 0.01, *** *p* < 0.001 versus non-Tg group on the same diet; ^#^*p* < 0.05 versus control fed of the same genotype.

At the age of 15–16 months, a significant (*p* < 0.05–0.001) increase in the number of activated microglia were detected in the hippocampus of 3xTgAD mice on either a control or high-fat diet when compared with non-Tg mice. A high-fat diet increased the number of activated microglia at the age of 3–4 months in 3xTgAD mice and at the age of 15–16 months in non-Tg mice (Fig. 7). The activated microglia were found in the same region of the hippocampus (dorsal subiculum) as to where the Aβ plaques were detected in the 3xTgAD mice (Fig. 6A).

## 4 Discussion

The present study is the first to assess longitudinally the effect of a high-fat diet in both control non-Tg and 3xTgAD mice, and to simultaneously measure cognitive function and pathology. We demonstrate that a high-fat diet increases the onset and severity of memory deficits in 3xTgAD mice. The effects of a high-fat diet on cognition in 3xTgAD mice are independent of an effect on AD neuropathology as no difference was observed in the deposition of Aβ and tau. Furthermore, high-fat feeding also caused memory impairments in control non-Tg mice.

Overconsumption of diets high in saturated fats is a common problem in developed countries and is likely the primary cause of obesity. Obesity is associated with an increased risk of several peripheral diseases but recent evidence suggests that obesity can also affect the brain. Midlife obesity and consumption of diets high in fat are linked to a greater risk of AD in humans (Beydoun et al., 2008; Eskelinen et al., 2008; Fitzpatrick et al., 2009; Grant, 1999; Gustafson et al., 2003; Hassing et al., 2009; Kalmijn et al., 1997; Kivipelto et al., 2005; Laitinen et al., 2006; Luchsinger et al., 2002; Profenno et al., 2009; Rosengren et al., 2005; Whitmer et al., 2005, 2007, 2008). Several studies have shown that obesity can also affect the brain in nondemented subjects and is linked with structural abnormalities, such as reduced brain and hippocampal volume, atrophy (e.g., temporal lobe), and white matter lesions (Gazdzinski et al., 2008; Gustafson et al., 2004a, 2004b; Jagust et al., 2005; Ward et al., 2005). Furthermore, central obesity has been linked with poorer cognition in elderly nondemented individuals (Jeong et al., 2005). These studies in obese humans suggest that increased adiposity can influence key areas in the brain that regulate memory and are affected in AD.

To date most studies on the effect of a high-fat diet on memory in rodents have assessed spatial memory only and have usually evaluated memory in one behavioral test. The present study used a battery of tests to assess the effect of high-fat consumption on spatial and nonspatial memory in mice. Regardless of the test used a high-fat diet impaired both spatial and nonspatial memory in non-Tg and 3xTgAD mice. In some behavioral tests a high-fat diet had a rapid effect on memory, although in other tests an effect was apparent at a later time point. A difference in the timing of the detrimental effect of a high-fat diet on cognition has been reported previously and is dependent on the use of spatial versus nonspatial memory tests (Kanoski and Davidson, 2011). These data therefore suggest that different modes of memory are differentially sensitive to the effects of a high-fat diet. Although the present study demonstrates that a high-fat diet caused a rapid but also long-lasting impairment in cognition some of these effects, especially in the non-Tg mice, were transient although this was not observed in all behavioral tests. The transient nature of the high-fat diet induced memory impairment is currently not understood. However, overall these data demonstrate that the choice of timing and the type of behavioral test used is therefore important when assessing the cognitive effects of high-fat consumption in mice.

In the present study the detrimental effect of a high-fat diet on memory in 3xTgAD mice was not associated with a change in the extent of Aβ and tau deposition. In support, not all studies reporting a worsening of memory in AD mice fed a high-fat diet have observed an effect on pathology (Herculano et al., 2013; Phivilay et al., 2009). However, several studies have shown that high-fat feeding can increase soluble Aβ and/or Aβ plaques in the brain of AD mice (Ho et al., 2004; Julien et al., 2010; Maesako et al., 2012a; Pedrini et al., 2009). Aβ oligomers are currently thought to be the key species involved in Aβ toxicity (Lesne et al., 2006; Shankar et al., 2008; Walsh et al., 2002) but in the present study no change in expression of soluble oligomers was detected in the hippocampus of 3xTgAD mice on a high-fat diet at any time point. High-fat consumption also caused memory deficits in control non-Tg mice, which do not present with Aβ or tau neuropathology. Furthermore, intermittent fasting has been shown to reduce cognitive deficits in 3xTgAD mice without effecting Aβ and tau levels (Halagappa et al., 2007). Thus, the effects of a high-fat diet on cognition in the present study are therefore likely not because of changes in AD-related neuropathology.

High-fat feeding caused an increase in body weight to the same extent in non-Tg and 3xTgAD mice. However, after the age of 7–8 months 3xTgAD mice on a high-fat diet had higher epididymal adipose mass compared with non-Tg control mice suggesting that the metabolic response to excess calories is different in the 3xTgAD mice. The present data also confirms a previous study showing that early in life 3xTgAD mice on a control diet weigh more and have higher food consumption than non-Tg controls (Knight et al., 2012), although food intake was not assessed here. This increased body weight shown here was not because of greater adipose tissue deposition, at least in the epididymal depots although other visceral depots and subcutaneous adipose remains to be assessed. It is likely therefore that other factors contribute to the greater body weight as 3xTgAD mice have longer body lengths and higher spleen mass (unpublished data). Altered metabolism has also been demonstrated in the 3xTgAD mouse as after the age of 12 months, these mice are no longer heavier than the control non-Tg mice even though food intake is still increased, an observation that might be because of a higher metabolic rate (Adebakin et al., 2012; Knight et al., 2012). However, it is likely that body weight and/or obesity per se may not be the main cause for cognitive deficits as memory deficits in high-fat fed AD mice can be reduced (with an antioxidant) without an effect on body weight (Herculano et al., 2013). Furthermore, when body weight is normalized after the high-fat diet is replaced with control diet, AD mice still experience cognitive deficits (Maesako et al., 2012b). Exercise is also more effective than diet control at reducing cognitive impairment in high-fat fed AD mice, even though exercise only induced a minor reduction in body weight (Maesako et al., 2012b). These data therefore suggest that long lasting metabolic consequences of high-fat feeding rather than body weight and/or adiposity are responsible for a reduction in cognition in mice.

The mechanisms responsible for the effect of a high-fat diet on memory remain unknown, although there are several possibilities. Neuroinflammation and oxidative stress are pathologic features of AD that are proposed to play a key role in the disease pathogenesis (Johnston et al., 2011; Verri et al., 2012; Wyss-Coray and Rogers, 2012). Microglia are the brain-resident “immune” cells and are proposed to play a key role in many neurodegenerative conditions and particularly in AD (Wyss-Coray and Rogers, 2012). Microglial activation was increased in response to a high-fat diet seen here in both 3xTgAD and non-Tg control mice, and also in other mouse models of AD (Herculano et al., 2013). Vascular inflammation might also be important in the effects of obesity on cognition as diets high in fat also increase expression of vascular inflammatory markers in AD mice (Herculano et al., 2013). Furthermore, when AD transgenic mice (APP23) are crossed with an obese mouse model (*ob/ob*) the resulting offspring (APP^+^-*ob/ob*) show worse cognitive deficits and vascular inflammation that appear before significant Aβ deposition (Takeda et al., 2010). Oxidative stress is also a key feature of AD and can be increased in response to dysregulated inflammation. Heighted oxidative stress is observed after short-term high-fat feeding in both AD and control mice and antioxidants have been shown to be reduce the memory deficits in AD mice fed a high-fat diet (Herculano et al., 2013).

The data in the present study indicate that high-fat diets impair memory in control and AD mice. It remains to be determined if the same or different mechanisms impact on memory in control and 3xTgAD mice, though the underlying mechanisms do not involve exacerbation of Aβ and tau pathology.

## Disclosure statement

The authors declare no conflicts of interest.

## Figures and Tables

**Table 1:** Epididymal fat weight of 3xTgAD and non-Tg mice in response to a high-fat diet

	Non-Tg		3xTgAD	
	Control	High-fat	Control	High-fat
3–4 mo	0.50 ± 0.04	1.46 ± 0.12^a^	0.37 ± 0.02	1.00 ± 0.09^a,^^c^
7–8 mo	0.55 ± 0.08	1.62 ± 0.09^a^	0.50 ± 0.04	2.51 ± 0.36^a,^^b^
11–12 mo	0.73 ± 0.11	1.50 ± 0.09	0.54 ± 0.05	4.75 ± 0.59^a,^^c^
15–16 mo	1.21 ± 0.14	1.35 ± 0.27	0.67 ± 0.09	5.56 ± 0.85^a,^^c^

Mice were maintained on a high-fat or control diet and separate groups of mice were monitored until the age of 3–4, 7–8, 11–12, or 15–16 months when epididymal fat pad weight (g) was assessed. Data are mean ± SEM, n = 6–12/group. ^a^*p* < 0.001 versus control fed mice of same genotype and ^b^*p* < 0.01, ^c^*p* < 0.001 versus non-Tg mice on the same diet. Two-way ANOVA with Bonferroni post hoc analysis. Key: ANOVA, analysis of variance; SEM, standard error of the mean.

**Table 2:** A high-fat diet had no effect on Alzheimer's disease neuropathology in 3xTgAD mice

	Cortex		Hippocampus		Amygdala	
	Control	High-fat	Control	High-fat	Control	High-fat
Aβ						
Plaque burden						
3–4 mo	nd	nd	nd	nd	nd	nd
7–8 mo	nd	nd	nd	nd	nd	nd
11–12 mo	nd	nd	680 ± 183	585 ± 264	nd	nd
15–16 mo	nd	nd	2713 ± 458	6177 ± 1845	nd	nd
Oligomers (nmol/L)						
3–4 mo	—	—	2.7 ± 0.4	3.9 ± 0.4	—	—
7–8 mo	—	—	4.2 ± 0.4	3.1 ± 0.2	—	—
11–12 mo	—	—	3.6 ± 0.4	3.1 ± 0.3	—	—
15–16 mo	—	—	3.8 ± 0.5	4.1 ± 0.4	—	—
Tau						
3–4 mo	nd	nd	nd	nd	nd	nd
7–8 mo	nd	nd	1 ± 1	nd	2 ± 2	1 ± 1
11–12 mo	nd	nd	7 ± 1	8 ± 3	2 ± 1	1 ± 1
15–16 mo	nd	nd	8 ± 6	3 ± 1	9 ± 4	12 ± 5

3xTgAD mice were maintained on a control or high-fat diet for those aged 3–4, 7–8, 11–12, and 15–16 months. Immunohistochemistry for Aβ or hyperphosphorylated tau was performed using 6E10 and AT8 antibodies, respectively. The average number of cells per section positive for tau were counted in the cortex, hippocampus, and amygdala. The extracellular Aβ plaque burden in the hippocampus was assessed and data are expressed as average plaque area/section (μm^2^). The expression of Aβ oligomers was analyzed by ELISA in the hippocampus. Data are mean ± SEM, n = 6–12/group. Key: Aβ, amyloid beta; ELISA, enzyme-linked immunosorbent assay; nd, none detected; —, not analyzed; SEM, standard error of the mean.
